# Editorial: Moral emotion, moral cognition, and (im)moral behavior in the workplace

**DOI:** 10.3389/fpsyg.2023.1232652

**Published:** 2023-08-25

**Authors:** Peixu He, Chuangang Shen, Hongdan Zhao, Cuiling Jiang

**Affiliations:** ^1^Business School, Huaqiao University, Quanzhou, China; ^2^School of Management, Shanghai University, Shanghai, China; ^3^Department of Management, KEDGE Business School, Talence, France

**Keywords:** moral emotion, moral cognition, moral behavior, immoral behavior, organizational behavior

## Introduction

Workplace moral behavior (e.g., pro-social behavior and organizational citizenship behavior) and immoral behavior (e.g., interpersonal abusive behavior, deviant/counterproductive behavior, and unethical pro-organizational behavior) have received substantial attention over the past decades (see for example Podsakoff et al., 2009; Dufy et al., 2012; Moore et al., 2012, 2018; Bolino and Grant, 2016; Chen et al., 2016; He et al., 2017, 2020, 2023a; Organ, 2018; Mishra et al., 2021). The existing research has provided us with knowledge on the antecedents and consequences of workplace moral and immoral behaviors (He et al., 2021). The most common framework that has been widely adopted to study the moral behavior has been the cognitive approach (Bandura, 1986, 1999), with rich evidence demonstrating that emotion and cognition are the two core elements which generate and influence workplace (im)moral behavior. However, we still have little knowledge on the emotional/cognitive processes or integrative moral emotion-cognition mechanisms related to workplace moral or immoral behavior. For example, it is unclear how exhibiting (im)moral behavior in the workplace would impact the perpetrator's and the third-party observer's emotions, thoughts, feelings, and subsequent behavior (Greenbaum et al., 2020). Besides, the question of whether ethical/unethical leader behavior would trigger a “trickle-down effect” is underdeveloped (Ogunfowora et al., 2022). In addition, when, how, and why the ethical employees (the so-called “good soldiers”) engage in workplace immoral behavior, and vice-versa, calls for further studies (He et al., 2023b).

The main goals of this Research Topic are to extend and enrich the research on workplace (im)moral behavior, offering the diverse insights to the individual emotion-centric models and social cognitive process models, exploring the individual dynamic moral emotions and moral cognition before and after the conduct of workplace (im)moral behavior, and clarifying the respective boundary conditions. Totally, we have accepted 28 papers for publication in the Frontiers in Psychology, which deal with different aspects of workplace (im)moral behavior, exploring mechanisms of these behaviors from the perspectives of moral cognition and moral emotion, and investigating the relevant antecedents and outcome variables. Some studies examined positive leadership (such as self-sacrificial leadership, responsible leadership, and moral leadership) and its impacts on subordinate performance and behavior (such as task performance, employee knowledge sharing behavior, and innovation behavior). We also included articles that investigate immoral behaviors in the organizations such as abusive supervision and counterproductive behavior. These studies have systematically examined the role of immoral behavior in the organizations from both moral cognition and emotion perspectives, which provide a complete understanding of the mechanisms underlying the immoral behavior. Moreover, we included one systematic review of executives' unethical behavior. Here, we provide an overview of the articles that have been included in this Research Topic.

## Overview of the articles in this Research Topic

The study “Linking Ethical Leadership to Followers' Knowledge Sharing: Mediating Role of Psychological Ownership and Moderating Role of Professional Commitment” was conducted by Saeed et al., which examines the impact of ethical leadership on knowledge sharing through the social learning theory. In this study, psychological ownership serves as a mediating variable and professional commitment serves as a moderating variable. The data was collected from employees of 307 Pakistani listed companies. The findings reveal that ethical leadership had a positive effect on knowledge sharing via psychological ownership which was buffered by professional commitment, enhancing the understanding in the field of leadership and knowledge management by identifying the role of ethics.

What about the relationship between responsible leadership and unethical pro-organizational behavior? In their work “Standing in Customers' Shoes: How Responsible Leadership Inhibits Unethical Pro-Organizational Behavior,” Cheng, Guo, et al. employ social information processing theory and social learning theory to build a moderated mediation model. Based on the sample of 557 participants, the results suggest that responsible leadership could inhibit unethical pro-organizational behavior and customer-oriented perspective taking partially may mediate the negative link between responsible leadership and unethical pro-organizational behavior.

In their work “The Impact of Moral Leadership on Physical Education Teachers' Innovation Behavior: The Role of Identification with Leader and Psychological Safety,” Chen J. et al., have used the sample of 287 teachers to examine the relationship between moral leadership and innovation performance.

The article “Servant Leadership Behavior at Workplace and Knowledge Hoarding: A Moderation Mediation Examination,” written by Zada S. et al. utilized social learning theory and data from 347 employees in 56 teams to investigate the relationship among servant leadership, psychological safety, and knowledge hoarding. The findings suggest that servant leadership negatively affects knowledge hoarding by positively influencing psychological safety, and mastery climate moderated the relationship.

The study “How Classy Servant Leader at Workplace? Linking Servant Leadership and Task Performance During the COVID-19 Crisis: A Moderation and Mediation Approach” by Zada M. et al., examined the impact of servant leadership on the task performance of employees in virtual working environments during the COVID-19 crisis. Drawing on the conservation of resources theory, the findings revealed that servant leadership is positively related to task performance in a virtual environment during crises. The study also found that psychological empowerment partially mediates this relationship, and perceived supervisor support positively moderates the relationship between servant leadership and task performance.

In their work “The Influence of Self-Serving Leadership on Deviant Behaviors in the Workplace: A Moderated Mediation Model,” Liu L. et al. have examined one type of destructive leadership, the self-serving leadership. Drawing from the social identity theory and 377 survey data via a three-wave time lagged design, the findings showed that self-serving leadership may induce employees' deviant behavior, organizational identification partially mediates self-serving leadership and employees' deviant behavior, and employees' moral identity negatively moderates the relationship between self-serving leadership and employees' organizational identification.

The study “Executives' unethical behavior with directions for future research,” is written by Zhu et al.. This bibliometric analysis has reviewed 428 articles published between the years 2000 and 2020 on executives' unethical behavior in the emerging markets.

In their work “The influence of leader–signaled knowledge hiding on tourism employees' work withdrawal behavior: A moderated mediating model,” Xu et al. have used conservation of resources theory to build a moderated mediation model with emotional exhaustion as a mediating variable and supervisor-subordinate guanxi as a moderating variable. The data was collected from 440 tourism employees. The results show that leaders' knowledge hiding is positively linked to employees' withdrawal behavior and emotional exhaustion.

Regarding the relationship between self-sacrificial leadership and employees' knowledge sharing, they study “How Does Self-Sacrificial Leadership Foster Knowledge Sharing Behavior in Employees? Moral Ownership, Felt Obligation and Supervisor-Subordinate Guanxi,” conducted by Su et al., employed the social cognitive theory and social exchange theory to explain how, when and why self-sacrificial leaders may trigger knowledge sharing. Totally, 481 pair sample has been used to test the theoretical model. The results showed that good guanxi between employees and their leaders, could lead employees to better understand leaders' self-sacrificial behavior and engage in knowledge sharing.

The study entitled “Does Self-Sacrifice Make Me Great? Research on the Relationship Between Employee Conscientiousness and pro-Social Rule Breaking” is written by Liu X. et al.. Drawing upon purposeful work behavior theory and using two-wave time lagged design, data was collected from 216 employee-supervisor dyads. The findings showed that duty orientation and achievement orientation have opposite relationship to pro-social rule breaking. Furthermore, job autonomy strengthens the positive effect of duty orientation and the negative effect of achievement orientation on pro-social rule breaking.

We have included another contribution related to employee consciousness. In their work “When Do Coworkers' Idiosyncratic Deals Trigger Social Undermining? – The Moderating Roles of Cored Self-Evaluations and Conscientiousness,” Wang and Ma used social comparison theory and a sample of 331 employees, to examine the interaction between perceptions of coworkers' receiving idiosyncratic deals and respective deprivation. The findings showed the moderating role of conscientiousness, which may weaken the relationship between deprivation and social undermining.

They study “When and How Workplace Helping Promotes Deviance? An Actor-Centric Perspective,” conducted by Zhang H. et al., examined the impacts of three helping behaviors (caring, coaching, and substituting helping) on helpers themselves. Drawing from the resource conservation theory and using a three-wave time-lagged design, the findings suggested that caring and coaching were more negatively associated with emotional exhaustion and helpers of these two styles were less likely to adopt subsequent deviant behaviors. This study has also identified the role of extrinsic career goals in the direct relationship between the three helping behavior and emotional exhaustion.

The contribution “A Contingency Perspective of Pro-Organizational Motives, Unethical Pro-Organizational Behavior, and Organizational Citizenship Behavior,” written by Cheng, Hu, et al., uses the data collected from 218 salespeople in the internet technology service company and develops a contingent model to show how moral identity and impression management motives could moderate the links between pro-organizational motives, unethical pro-organizational behavior, and organizational behavior.

The article “Status Competition and Implicit Coordination: Based on the Role of Knowledge Sharing and Psychological Safety” written by Xiao J. et al., has examined the implicit coordination from the perspective of team mentality, and discussed the incentive mechanism of status competition by using knowledge sharing as a mediating variable and psychological safety as a moderating variable. The empirical study has used a sample of 367 employees from 44 companies. The findings revealed that prestige-type status competition was positively related to implicit coordination, while dominant-type status competition was negatively related to implicit coordination. Furthermore, knowledge sharing mediated the relationship between both types of status and implicit coordination, and psychological safety enhanced both relationships.

In the study “Research on the Relationship Between High-Commitment Work Systems and Employees' Unethical Pro-Organizational Behavior: The Moderating Role of Balanced Reciprocity Beliefs,” Zhang M. et al. uncovered the situational factors that influence employees' unethical pro-organizational behavior to repay the organization. Based on the social exchange theory, the authors used multisource data from 139 human resource managers and 966 employees to examine why and how high-commitment work systems affect employees' unethical pro-organizational behavior. The findings revealed that high-commitment work systems promote employees' unethical pro-organizational behavior through relational psychological contract. Besides, the findings showed that balanced reciprocity beliefs strengthen the positive relationship between relational psychological contract and employees' unethical pro-organizational behavior.

Zhang Z. et al. conducted the study “Does Technostress Increase R&D Employees' Knowledge Hiding in the Digital Era?” and introduced work exhaustion as a mediating variable for exploring how five sub-dimensions of technostress impact R&D employees' knowledge hiding. The authors have used job demand-resource theory to examine technostress—an antecedent of knowledge hiding. The sample of 254 participants was collected through a two-stage survey. The findings showed that techno-invasion, techno-insecurity, and techno-complexity were significantly and positively associated with work exhaustion. Furthermore, work exhaustion mediated the relationship between aforementioned three variables and knowledge hiding, while workplace friendship negatively moderated the association between techno-invasion, techno-insecurity and work exhaustion, reducing the emergence of knowledge hiding, while it also positively moderated the association between techno-complexity and work exhaustion.

The study “Workplace Suspicion, Knowledge Hiding, and Silence Behavior: A Double-Moderated Mediation Model of Knowledge-Based Psychological Ownership and Face Consciousness” conducted by Wu et al. examined the relationship between workplace suspicion and employees' silence, as well as the mediating role of knowledge hiding from a colleague's perspective. Drawing from resource conservation theory and self-regulation theory, data of 303 pair sample from 23 companies in China was collected through three-waves questionnaire method. The results revealed that workplace suspicion positively influenced employees' silence via knowledge hiding, and knowledge-based psychological ownership strengthened the mediating effect. However, face consciousness weakened the positive impact of workplace suspicion on knowledge hiding.

The study “Impacts of Corporate Social Responsibility on Employees' Mental Fatigue” written by Zheng et al. uses 176 valid responses to examine whether employees' personal ethics and perceptions of corporate hypocrisy can be beneficial for reducing employees' mental fatigue. The theories used to develop the hypotheses are stakeholder theories and sociological theories. The findings showed that employees' mental fatigue reduces when internal or external corporate social responsibility (CSR) has a positive impact on employees' altruistic choice and employees' mental fatigue increases when CSR has a negative effect on ethical egoism.

Existing research seldom employs a bottom-up perspective to examine how employees can reduce abusive supervision. In their work, Jiang et al. conducted the study of “Benefits of Non-Work Interactions with Your Supervisor: Exploring the Bottom-Up Effect of Employee Boundary Blurring Behavior on Abusive Supervision” and explored how employees' boundary blurring behavior can prevent themselves from being abused by supervisors. Drawing from the self-disclosure theory, authors have used a scenario-based experimental study and a multi-wave field study to find out that employees' boundary blurring behaviors inhibit the emergence of abusive supervision through the mediating effect of supervisor liking toward the employee.

In the article “Appearing competent or moral? The role of organizational goals in the evaluation of candidates,” Fousiani et al. explored the effect of morality and competence in recruiters' hiring decisions. The authors used the Big Two theoretical framework to examine how instrumental or relational goals of organizations might influence the importance of morality or competence of candidates during the hiring process. The authors conducted three studies (Study1, *n* = 260; Study2, *n* = 318; Study3, *n* = 394) to test the proposed hypotheses. The findings showed that the primacy effect of morality might hold when organizational goals are relational but might get reversed when organizational goals are instrumental. They also found that perceived appropriateness of a candidate positively affects hiring recommendations.

The contribution of Carminati and Héliot, titled “Between Multiple Identities and Values: Professionals' Identity Conflicts in Ethically Charged Situations,” adopted a qualitative approach. Forty-seven semi-structured interviews had been conducted among doctors and nurses working for the English National Healthcare Service. The findings showed that micro processes such as cognitive and emotional perspective taking, plus identifying with the other may trigger identity conflict.

The study “Person–Job Misfit: Perceived Overqualification and Counterproductive Work Behavior” by Khan et al. examined the relationship between perceived overqualification and counterproductive work behavior (CWB) among textile sector employees, considering job boredom as a mediator and job crafting as a moderator. The findings showed a positive relationship between perceived overqualification and CWB. Furthermore, the study found that job boredom mediated the relationship between perceived overqualification and CWB, and job crafting moderated the positive association between perceived overqualification and job boredom.

The article by Shen et al. titled “How I Speak Defines What I Do: Effects of the Functional Language Proficiency of Host Country Employees on Their Unethical Pro-Organizational Behavior” investigates the relationship between functional language proficiency (i.e., English) and unethical pro-organizational behavior of host country employees in multinational corporations (MNCs). The authors used data from 309 full-time host country employees to test their predictions guided by social identity theory. The findings suggested that host country employees' functional language proficiency enhances their unethical pro-organizational behavior through their linguistic group identification and moral disengagement.

The article “How does leaders' information-sharing behavior affect subordinates' taking charge behavior in public sector? A moderated mediation effect,” is written by Liu J.-N. et al.. Drawing from planned behavior theory and 200 civil servants' data, the findings showed that public sector leaders' information-sharing behavior is positively related to their subordinates' taking charge behavior, and public service motivation mediates this relationship. The results also found emotional trust strongly moderates the effect of leaders' information-sharing behavior on subordinates' taking charge behavior.

In their work “Is not workplace gossip bad? The effect of positive workplace gossip on employee innovative behavior,” Dai et al. have examined the role of positive workplace gossip through a moderated mediation model. Data from 327 employees in the Pearl River and Yangtze River Delta regions of China was collected to test the theory model. The results showed that positive workplace gossip may promote employee innovation.

The article “How Daily Supervisor Abuse and Coworker Support Affect Daily Work Engagement” by Wang and Tang aimed to investigate the impact of daily supervisor abuse and coworker support on daily work engagement. Drawing from the conservation of resources (COR) theory, the study utilized a daily diary approach and collected data from 73 employees during five consecutive days in China. The results showed that daily abusive supervision had a negative impact on daily work engagement and that daily negative emotions mediated this relationship. Coworker support had a cross-level moderating effect between daily abusive supervision and daily negative emotions.

The study “Influence of Crowd-sourcing Innovation Community Reference on Creative Territory Behavior” by Xiao W. et al. investigates the impact of crowdsourcing innovation community reference on creative territory behavior from the perspective of reference group theory. A two-stage survey has been used and 524 valid responses were collected. The results suggested that the crowdsourcing innovation community reference influences members' impression management behavior and then inhibits creative territory behavior. Interestingly, there are different community reference effects among members of different community age groups. These findings contribute to understanding the influence of the crowdsourcing innovation community on crowd participation decision-making and suggest implications for exploring the cooperation mechanism of crowdsourcing innovation.

In another perspective, the article “Why Does Leader Aggressive Humor Lead to Bystander Workplace Withdrawal Behavior?—Based on the Dual Path Perspective of Cognition Affection,” Chen H. et al. examined the relationship between leader's aggressive humor and bystander's workplace withdrawal behavior. The study used the Cognitive-Affective Personality System Theory and collected data from 443 employees and their direct supervisors in the Chinese enterprises. The results showed that leader-aggressive humor positively affected bystander affective rumination and bystander workplace anxiety, which in turn mediated the relationship between leader-aggressive humor and bystander workplace withdrawal behavior. Additionally, organization-based self-esteem moderated the indirect impact of leader-aggressive humor on bystander workplace withdrawal behavior through bystander affective rumination and bystander workplace anxiety.

## Contributions

This Research Topic has made the following contributions. First, in this Research Topic, scholars attempted to conduct research from different perspectives and methodologies on the “good” behaviors (e.g., organizational citizenship behaviors) and negative behaviors (e.g., workplace gossip), which have enriched the research on the “dark sides” of workplace positive behavior and the “bright sides” of workplace negative behavior. For example, the study of Dai et al. have explored the effect of positive workplace gossip on employee's innovative behavior; Zhang H. et al. have studied when and how workplace helping promotes workplace deviance from an actor-centric perspective; Zhang M. et al. have explained the dark side of high-commitment work systems and linked it to employees' unethical pro-organizational behavior. These findings point out the importance to review and manage workplace (un)ethical/(im)moral behaviors with a balanced but critical perspective.

Second, prior research has explored the relationship between various types of destructive leaders' behavior (e.g., abusive supervision) and workplace unethical/immoral behaviors such as counterproductive work behavior, and the relationship between positive leadership (e.g., ethical leadership) and employees' positive workplace behaviors such as innovative behavior and prosocial behavior. However, how positive leadership can help prevent unethical organizational behaviors requires further development. In this Research Topic, Cheng, Guo, et al. have examined how positive leadership can prevent employees' workplace unethical/immoral behaviors. This contribution has greatly enriched the current understanding of how responsible leadership is related to employees' unethical pro-organizational behavior. Furthermore, this study has explicitly explained the impacts of positive leadership and the important role that positive leadership can play in inhibiting and governing the negative behaviors of employees.

Third, this Research Topic focuses on the mechanisms by which relationships and interpersonal interactions between supervisors and subordinates and between coworkers influence each other's (un)ethical/(im)moral behaviors. We have included studies of top-down leadership behaviors and their impacts on employees' behaviors, and the studies of the bottom-up employees' behaviors and their impacts on leaders' behaviors. The (un)ethical/(im)moral behaviors in the workplace are examined through multiple directions. For example, Xu et al. have explored the influence of leader-signaled knowledge hiding on tourism employees' withdrawal behavior. Jiang et al. have advocated the benefits of employees' non-work interactions with their supervisors and verified the bottom-up effect of employee boundary blurring behavior on abusive supervision. Liu J.-N. et al. have investigated how leaders' information sharing behavior affect subordinates' taking charge behavior in public sector. Chen H. et al. have further studied the relationship between leader's aggressive humor and employee's withdrawal behavior from a bystander's perspective. Su et al. have demonstrated the impacts of leaders' self-sacrificial behaviors and supervisor-subordinate *guanxi* on employees' knowledge sharing. Wang and Tang have integrated daily supervisor abuse and coworker support in influencing employees' daily work engagement. These contributions point out that when managing leaders' or employees' workplace (un)ethical/(im)moral behaviors, one should not only look for the reasons and respective governance strategies from the actors' perspectives. In fact, the influence of others is an important source of the formation of individual behaviors in the workplace. Managers should improve the positive interpersonal interactions through building the healthy and meaningful relationship at the workplace, and reduce the contagion effects through lead by example, open communication, promotion of positive corporate culture, rewards on the ethical/moral behaviors, and so on.

Last, knowledge-related behaviors such as knowledge sharing (see for example Liu J.-N. et al.; Su et al.; Xiao J. et al.; Saeed et al.), knowledge hiding (see for example Xu et al.; Wu et al.; Zhang Z. et al.), and knowledge hoarding (see for example Zada S. et al.) have attracted increasing attention. The contributions have employed theoretical lens such as the conservation of resources theory, the self-regulation theory, the framework of “cognition-motivation-behavior,” and the psychological safety and psychological ownership perspectives, to examine the formation mechanism between knowledge sharing behavior and counterproductive knowledge behavior. In addition, these contributions have provided a systematic review related to the moral concern of knowledge hiding. Thanks to these studies, we are able to enrich the knowledge hiding research by diversifying the existing theoretical perspectives, realizing the interdisciplinary studies, introducing and integrating the moral ownership, moral self-regulation and other moral psychology, cognitive psychology and other disciplines in the theoretical perspectives, forming new insights to call for research on the novel knowledge hiding phenomenon (i.e., leader-signaled knowledge hiding). Furthermore, the contributions that have been included in this Research Topic have taken the lead in exploring how technostress increase R&D employee knowledge hiding in the digital era. These findings draw practitioners' attention to the ethical nature of knowledge hiding behaviors and the respective moral issues. This Research Topic offers practitioners with knowledge on how to better anticipate the harmfulness of immoral behavior and the dark sides of moral behavior, developing the knowledge governance practices from the perspectives of employees' moral emotion and moral cognition.

## Author contributions

All authors listed have made a substantial, direct, and intellectual contribution to the work and approved it for publication.
